# Fluoroscopy-assisted identification of the individual optimal antegrade entry point of the anterior column fixation corridor in pelvic and acetabular surgery: a novel perspective

**DOI:** 10.1186/s13018-025-06027-0

**Published:** 2025-07-09

**Authors:** Vedat ÖZTÜRK, Mustafa Gökhan BİLGİLİ

**Affiliations:** Department of Orthopedics and Traumatology, Bakırköy Dr. Sadi Konuk Training and Research Hospital, Tevfik Sağlam St. Number 11, Bakırköy, Istanbul, 34147 Türkiye

**Keywords:** Acetabulum surgery, Anterior column, Fixation corridor, Antegrade screw placement, Virtual study

## Abstract

**Introduction:**

This study aims to describe a novel axial fluoroscopic imaging technique for visualizing the anterior column fixation corridor (ACFC) of the acetabulum in the supine position, define the patient-specific optimal antegrade entry point (OAEP), and evaluate the feasibility of screw placement using this approach.

**Materials and methods:**

Pelvic computed tomography (CT) data from 500 healthy adults (250 men and 250 women) were collected. Using Fujifilm-Synapse 3D software, 3D reconstructions of the pelvis were created. Through fluoroscopy simulation, the axial view of the ACFC and OAEP was obtained for each individual. To simulate screw placement radiologically, a cylinder was placed through the OAEP, completely filling the corridor without protruding. The position was verified using both fluoroscopic simulations and three different CT sections (axial, coronal, sagittal). The corridor’s diameter (ACFC-R), length (ACFC-L), and the coronal plane inclination (CPI) and sagittal plane inclination (SPI) required for fluoroscopic visualization of the OAEP were measured in all pelvic models.

**Results:**

The axial view and patient-specific optimal antegrade entry point (OAEP) of the fixation corridor were successfully visualized in all pelvises. Radiological virtual screw placement was successfully performed in all models by visualizing the OAEP, enabling precise axial screw insertion through the corridor. The average ACFC diameters were 6.2 mm in females and 8.6 mm in males (*p* < 0.001); ACFC lengths were 116.8 mm in females and 122.5 mm in males (*p* < 0.001). The SPI was 14.3 degrees in females and 14.7 degrees in males (*p* = 0.263). The CPI was 35.5 degrees in females and 33.2 degrees in males (*p* < 0.001). Except for the SPI, statistically significant differences were observed in all parameters between genders.

**Conclusion:**

The axial fluoroscopic imaging technique enables accurate identification of patient-specific entry points and screw placement that fills the fixation corridor without boundary breach, potentially enhancing the precision and safety of anterior column fixation.

**Supplementary Information:**

The online version contains supplementary material available at 10.1186/s13018-025-06027-0.

## Introduction

Pelvic and acetabular fractures present significant challenges in orthopedic surgery due to their complex three-dimensional anatomical structure and proximity to critical vascular and neural structures [1]. Accurate stabilization and treatment of these fractures are crucial for ensuring functional recovery and maintaining the quality of life in patients [2]. Several fixation corridors have been described to achieve stable fixation in acetabular fractures such as supraacetabular, infraacetabular, posterior column, anterior column, and the recently defined both-column fixation corridor [3–5].

The placement of screws in these corridors must be performed with great precision to avoid damage to surrounding vital structures and to ensure stable fixation. Therefore, the development of screw placement techniques play a crucial role in enhancing surgical success [3, 6]. In recent years, computer-assisted navigation systems and patient-specific 3D navigation templates have been introduced to optimize the screw placement process. However, the high cost of these advanced systems and their limited availability in the operating room make them difficult to access [7, 8]. Fluoroscopic techniques, on the other hand, are widely used due to their practicality and broader accessibility. However, accurate screw placement using traditional fluoroscopic methods can be challenging due to the complex anatomical structure of the pelvis. Therefore, it is of paramount importance to develop reliable, practical, and specific techniques for screw placement under fluoroscopic guidance [3].

The aim of the study is to demonstrate step-by-step screw placement in the ACFC using the axial fluoroscopic imaging technique in the supine position, evaluate the feasibility of this approach, provide technical guidance for the fluoroscopic visualization of this corridor, and investigate anatomical differences between genders, offering surgical insights during procedures. Ultimately, it aims to contribute to the safer and more effective use of fluoroscopic methods in clinical practice.

## Primary and secondary outcomes

The primary outcomes of this study include the axial visualization of the Anterior Column Fixation Corridor (ACFC) and the patient-specific optimal antegrade entry point (OAEP), as well as the feasibility of using this axial view to successfully perform antegrade screw placement without breaching the corridor in both genders.

Secondary outcomes include the investigation of gender-specific anatomical variations and the analysis of measurements taken during the antegrade screw placement process.

These measurements include the diameter (R) and length (L) of the ACFC, as well as the analysis of the angulation required for the fluoroscopy machine in both the coronal plane (CPI) and the sagittal plane (SPI) to achieve axial fluoroscopic visualization of the ACFC in clinical practice.

## Material and method

### Study design and setting

This retrospective study was conducted at a Level 1 trauma center following approval from the local ethics committee. Also the study has been registered on ClinicalTrials.gov. All patient data were obtained from the hospital’s digital radiology archive records.

### Inclusion and exclusion criteria

Pelvic computed tomography (CT) scans obtained from the hospital’s digital radiology archive, with a slice thickness of 1 mm, accurately depicting the entire anatomy of the pelvis, showing evidence of complete bone maturation, and displaying no signs of fractures, deformities, previous orthopedic trauma, prior orthopedic surgery, rheumatologic sequelae, advanced osteoporosis, bone metastases, metabolic bone diseases, or other bone pathologies, from patients aged 18–65, were included in the study. CT scans that did not meet these criteria were excluded.

### Comprehensive description of study and measurements (Technical Video)

**Fig. 1:**
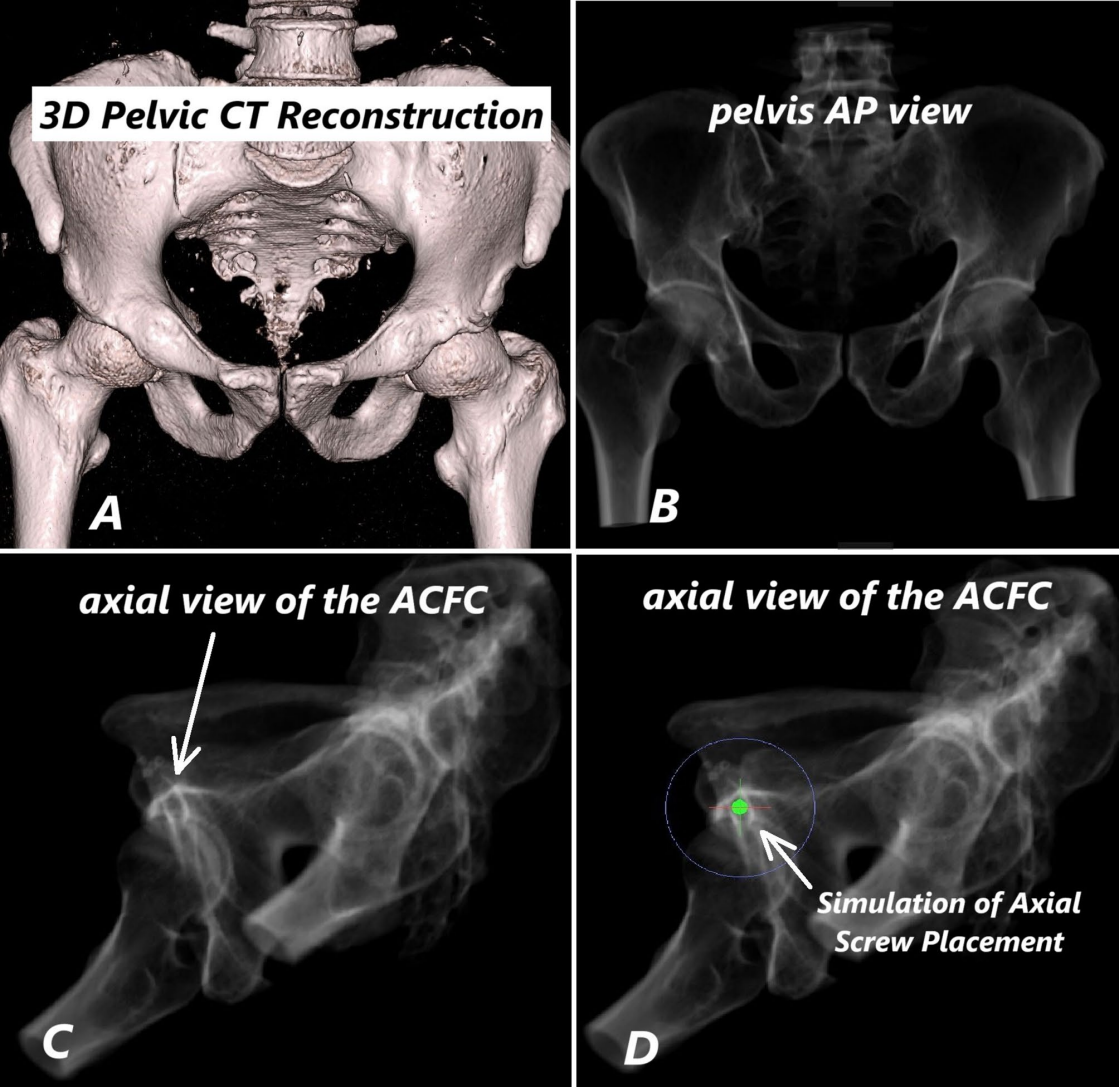
Synapse3D software-generated 3D pelvic reconstruction (**A**). Simulated AP X-ray image (**B**). Axial fluoroscopic view of the ACFC (**C**). Cylindrical screw model placed in the ACFC (**D**)

Fujifilm-Synapse3D software was used to simulate radiological screw placement. Pelvic CT images were first converted into 3D reconstructions (Fig. 1A), and then transformed into simulated fluoroscopic views (AP and axial) to visualize the Anterior Column Fixation Corridor (ACFC) (Fig. 1B–C).

**Fig. 2:**
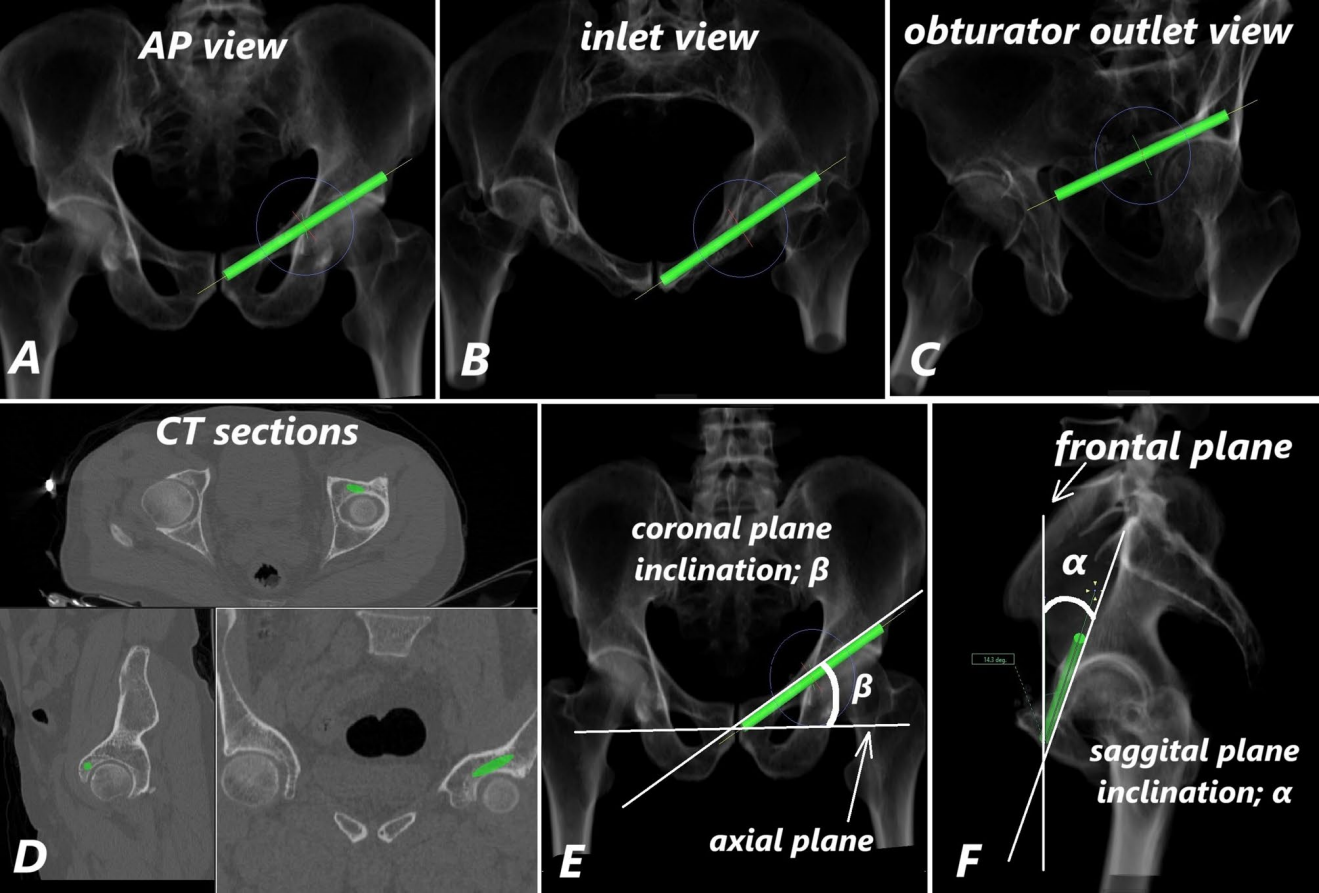
Fluoroscopic views (AP, inlet, obturator outlet) and axial, sagittal, coronal CT sections showing the screw within the ACFC without breaches (**A-D**). Measurement of fluoroscopic angles: CPI and SPI (**E-F**)

A cylindrical model representing the screw was placed into the ACFC in the axial view (Fig. 1D). The screw length was increased incrementally by 1 mm, and the screw diameter by 0.5 mm, to determine the largest possible dimensions without breaching the medial or lateral walls of the corridor or penetrating the acetabulum. Final screw positioning was verified using simulated fluoroscopic images in AP, inlet, and obturator outlet views (Fig. 2A–C), as well as axial, sagittal, and coronal CT slices (Fig. 2D).

Based on the optimally positioned screw, the ACFC length (L) and diameter (R) were measured. Additionally, coronal and sagittal inclination angles—CPI and SPI—were calculated relative to standard fluoroscopic planes (Fig. 2E–F), to facilitate intraoperative visualization of the ACFC during surgical procedures.

### Statistical analysis

IBM SPSS 26 software was used for statistical analysis. Descriptive statistical methods (min, max, and median) were used when evaluating the study data. The normality of continuous variables was assessed using the Kolmogorov–Smirnov test and graphical methods. The results of the normality tests indicated that none of the continuous variables followed a normal distribution (AGE: D = 0.0817, *p* < 0.001; SPI: D = 0.0538, *p* = 0.0015; CPI: D = 0.0763, *p* < 0.001; ACFC-R: D = 0.0881, *p* < 0.001; ACFC-L: D = 0.0616, *p* = 0.0429). Therefore, group comparisons were performed using the Mann–Whitney U test. To quantify the magnitude of differences, effect sizes (r) were calculated from Z values and interpreted using standard thresholds (**r = Z / √N**). Statistical significance was defined as *p* < 0.05.

## Results

### Descriptive data

A total of 500 participants were included in the study, with an equal gender distribution (250 females and 250 males). The overall mean age was 39.49 ± 14.34 years (range: 18–65), with the mean age in the female group being 39.63 ± 14.20 years (range: 18–65), and 39.35 ± 14.51 years (range: 18–65) in the male group (Table 1).

**Table 1:** Demographic characteristics and measurements by gender for patients undergoing radiological virtual screw placement

	SEX		Total (*n*; 500) mean ± sd, median (min-max)	*p*-value*	Z value	Effect size (*r*)	interpretation
	Female (*n*; 250) mean ± sd, median (min-max)	Male (*n*; 250) mean ± sd, median (min-max)					
AGE	39.6 ± 14.2, 40.0 (18–65)	39.3 ± 14.5, 40.0 (18–65)	39.4 ± 14.3,40.0 (18–65)	0.850	-0.189	0.008	negligible
SPI (°)	14.3 ± 6.6, 14.0 (3–36)	14.7 ± 5.6, 14.0 (0–35)	14.5 ± 6.1,14.0, (0–36)	0.263	-1.119	0.05	negligible
CPI (°)	35.5 ± 3.8,33.5 (22–45)	33.2 ± 6.1, 33.5 (13–45)	34.3 ± 5.2, 35 (13–45)	< 0.001	-4.806	0.215	small to moderate
ACFC-R (mm)	6.2 ± 1.2, 6.0 (4–10)	8.6 ± 1.2, 8.5 (5–11)	7.4 ± 1.7, 7.5 (4–11)	< 0.001	-15.350	0.686	large
ACFC-L (mm)	116.8 ± 6.5, 117 (102–135)	122.5 ± 6.8, 123.0 (103–148)	119.6 ± 7.2, 119 (102–148)	< 0.001	-9019	0.403	moderate to large

*Mann–Whitney U test,*** r = Z / √N (effect size for Mann–Whitney U test)***

***SPI;****Saggital plane inclination*, ***CPI;****Coronal plane inclination*, ***ACFC-R;****Anterior column fixation corridor radius*, ***ACFC-L;****Anterior column fixation corridor lenght*

### Axial fluoroscopic imaging of anterior column fixation Corridor (ACFC)

Our research revealed that the axial image and patient-specific optimal antegrade entry point (OAEP) of the ACFC could be successfully visualized in all 500 pelvic models. This finding directly demonstrates that axial view and OAEP of the ACFC can be visualized in all patient populations studied, regardless of gender.

### Overall measurements

The mean values of the measured parameters in the total population were as follows: ACFC Diameter (ACFC-R) 7.43 ± 1.72 mm (range: 4.0–11.0), ACFC Length (ACFC-L) 119.69 ± 7.28 mm (range: 102–148), Coronal Plane Inclination (CPI) 34.39° ± 5.23° (range: 13°–45°), and Sagittal Plane Inclination (SPI) 14.52° ± 6.17° (range: 0°–36°) (Table 1).

### Gender-specific anatomical variations

When gender-specific anatomical variations were examined, ACFC diameter and length were significantly greater in males, while CPI was higher in females. No significant gender-based differences were observed in SPI or age. Corresponding *p*-values, Z scores, effect sizes (r), and interpretations are presented in Table 1.

## Imaging of the anterior column fixation corridor (ACFC) and screw placement procedure

### Fluoroscopy setup for axial visualization of ACFC

**Fig. 3:**
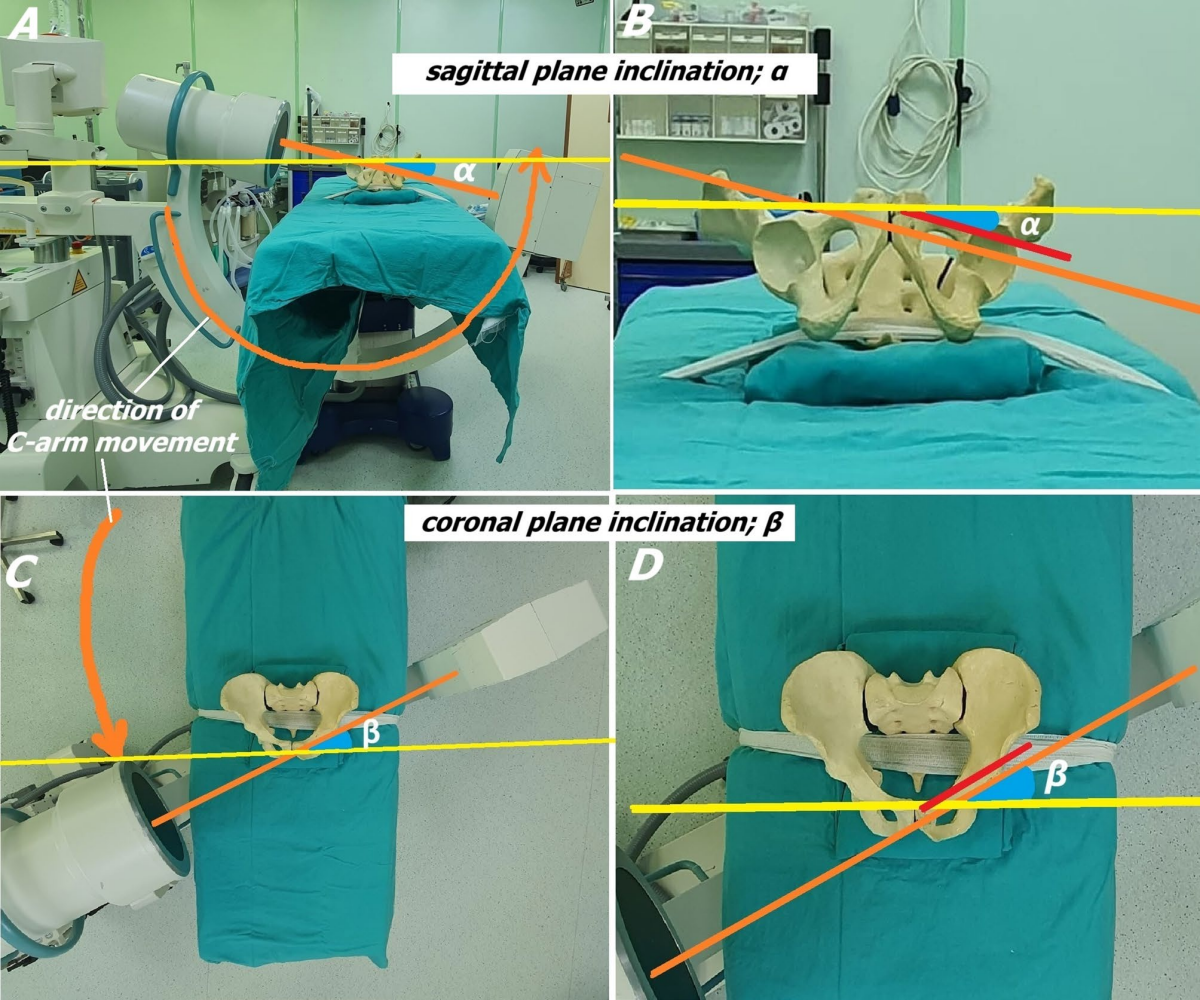
Posterior tilt of the C-arm aligning with the sagittal plane inclination of the ACFC (SPI; α) (**A**). The fluoroscopic imaging axis is represented by orange line segments, while the frontal plane in the supine position is shown as a yellow line segment (**A**, **B**). Axis of the ACFC; red line segment (**B**). Axial alignment of the fluoroscopic imaging axis with the coronal plane inclination (CPI; β) of the ACFC (**C**). Here, the fluoroscopic imaging axis is again represented by orange lines, and the axial plane in the supine position is indicated by a yellow line (**C**, **D**). Axis of the ACFC; red line segment (**D**)

Once the patient is placed supine on a radiolucent table, positioning must ensure that a true anteroposterior (AP) pelvic image is obtained fluoroscopically. This view serves as the baseline reference for subsequent fluoroscopic angulations. Ensuring this standard view is essential for accurate anatomical orientation and minimizing radiation exposure. The fluoroscopy unit is then aligned for axial visualization of the ACFC and positioned on the opposite side of the targeted screw placement area; for example, if the left ACFC is targeted, the machine is placed on the patient’s right side (Fig. 3).

Subsequently, to achieve axial visualization of the corridor, the imaging axis of the fluoroscopy is aligned roughly parallel to the axis of the ACFC (Fig. 3). Two important adjustments are made for this. The first adjustment involves tilting the C-arm posteriorly in the sagittal plane to mimic the posterior tilt of the ACFC relative to the frontal plane (SPI; α angles in Fig. 3A-B). Initially, the image intensifier of the fluoroscopy machine is positioned on the opposite side of the working area, and the C-arm is tilted posteriorly by approximately 15 degrees in the sagittal plane to match the inclination of the ACFC (Fig. 3A-B). The second adjustment ensures that the axial imaging axis of the fluoroscopy is aligned parallel to the axial axis of the ACFC in the coronal plane (CPI; β angles in Fig. 3C-D). For this, the symphysis pubis is first palpated, and the axial imaging axis of the fluoroscopy is aligned to pass through the symphysis pubis and run parallel to the coronal axis of the ACFC in the coronal plane. Since the ACFC has an approximate inclination of 35 degrees in the coronal plane, the fluoroscopy machine is similarly angled (Fig. 3C-D). The combination of these angular adjustments results in the axial fluoroscopic visualization of the ACFC.

Fine adjustments can be made in both positions to optimize the axial view (Note: The angles provided here are based on our clinical experience and are supported by the data obtained from the study, see Table 1).

### Description of fluoroscopic boundaries of the axial view of the fixation corridor: Falcon sign

**Fig. 4:**
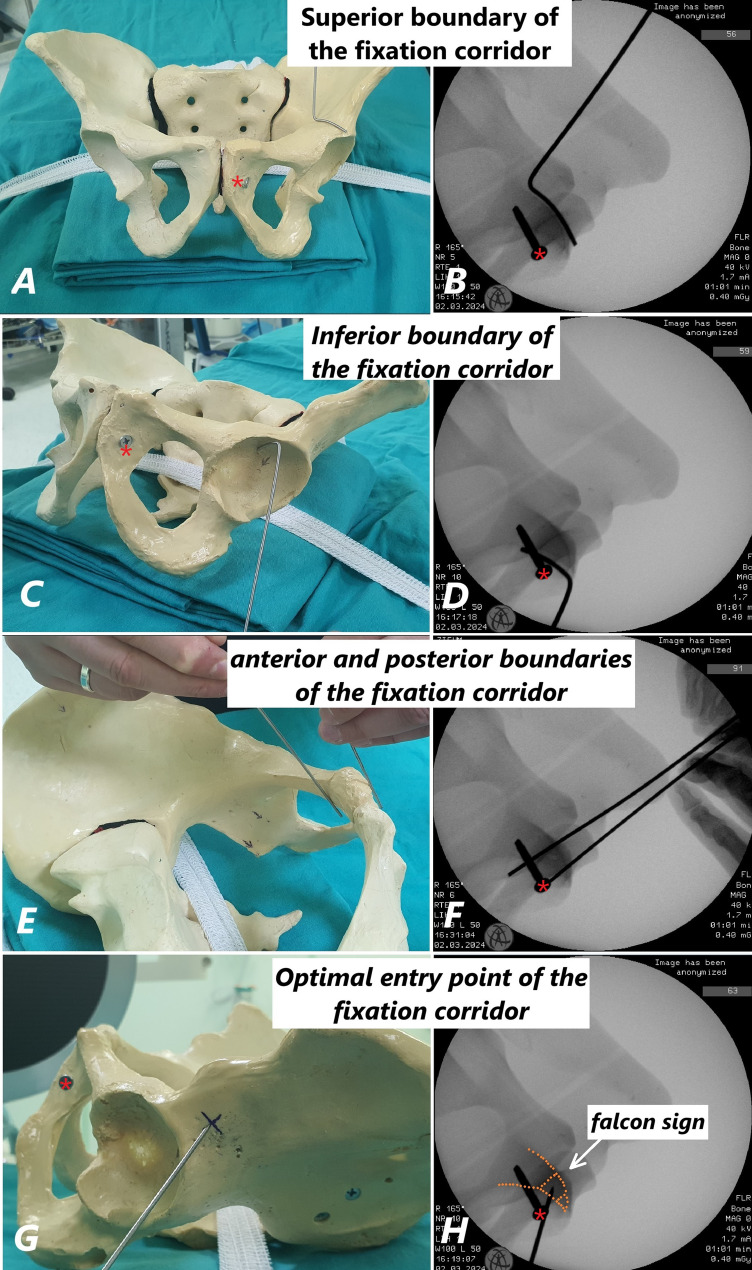
ACFC boundaries: ilio-pubic junction (**A**, **B**), acetabular roof (**C**, **D**), and anterior/posterior walls near the pubic symphysis (**E**, **F**). The ‘Falcon Sign’ shows the optimal entry point (**G**, **H**). (Note: The screw marked with a red asterisk (*) in both the sawbone model and fluoroscopic images is not related to the described technique. It is an ordinary screw used solely for symphysis pubis fixation in the sawbone model.)

Since the ACFC is located within the superior pubic ramus (SPR), the axial fluoroscopic view of the corridor is obtained by the intersection of the proximal and distal portions of the SPR. In the axial plane, the superior and inferior boundaries of the corridor are defined by the proximal part of the SPR. Proximally, the ilio-pubic junction forms the superior boundary (Fig. 4A-B), while the superior acetabular roof forms the inferior boundary (Fig. 4C-D). Distally, toward the pubic symphysis, the anterior and posterior walls of the SPR define the anterior and posterior boundaries of the corridor (Fig. 4E-F). This axial view also represents the patient-specific optimal entry point for antegrade screw placement, which is critical during surgery (Fig. 4G-H). Due to its resemblance to a specific shape, we have named this view the ‘Falcon Sign’ (Fig. 4H).

### Intraoperative technique

**Fig. 5:**
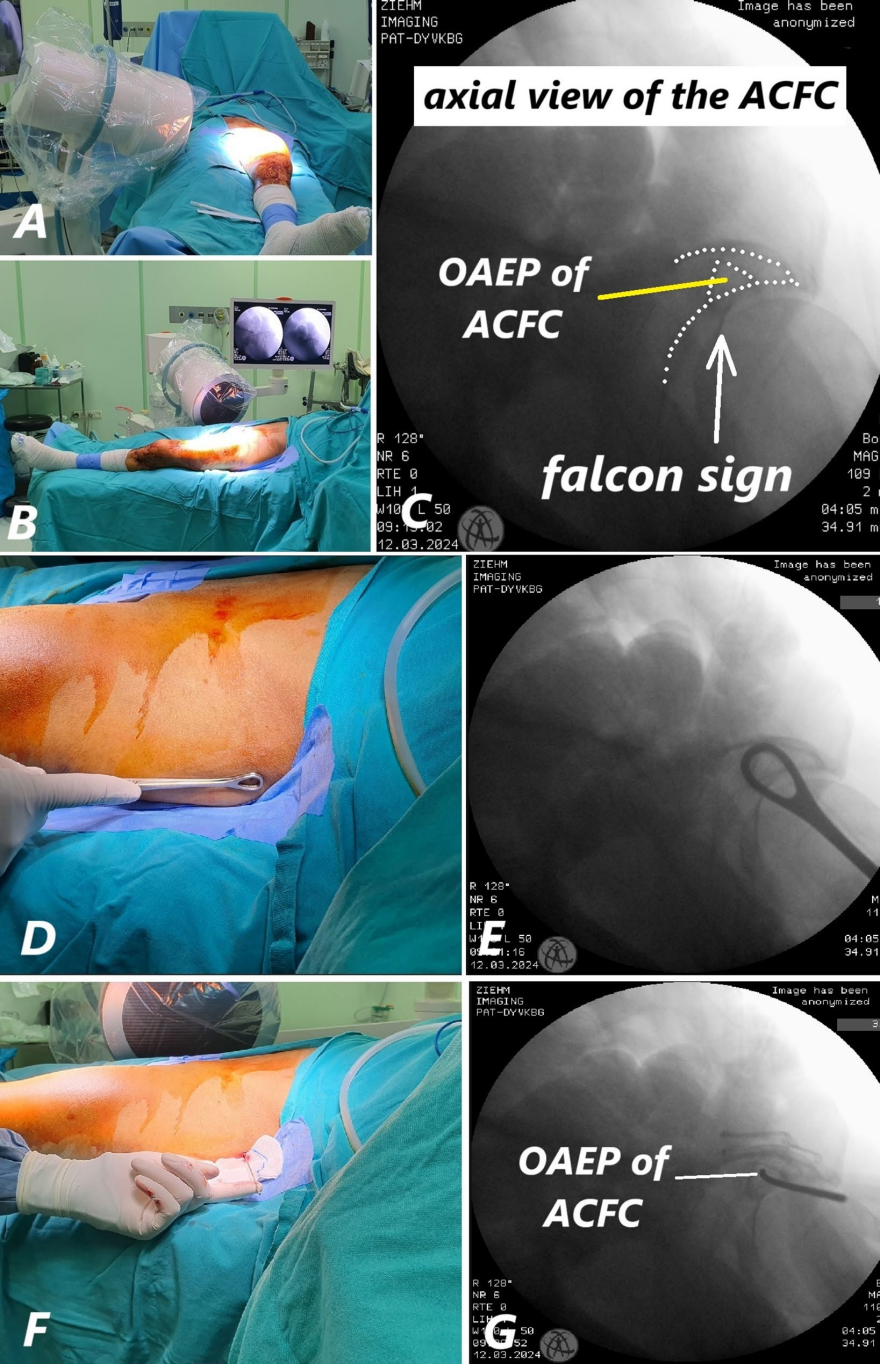
Axial view showing patient-specific OAEP (**A-C**). Skin marking with a radiopaque marker (**D**, **E**). K-wire placement guided by fluoroscopy (**F**, **G**)

As described in the technique above, with the patient in the supine position, the axial view of the ACFC (Falcon Sign) is obtained by properly positioning the fluoroscopy (Fig. 5A-C). This axial view also represents the patient-specific optimal antegrade entry point (OAEP) for screw placement, which is critical during surgery (Fig. 5C). Under fluoroscopic guidance, the optimal entry point is marked on the skin using a radiopaque marker (Fig. 5D-E). Next, a K-wire is placed into the center of the corridor under fluoroscopic control and lightly tapped into the bone cortex to prevent slippage (Fig. 5E-F). Once the optimal entry point is determined, the fluoroscopy is repositioned to display both the inlet and obturator outlet views. Using a drill, the K-wire is advanced into the corridor in a controlled manner. After confirming that the K-wire remains within the corridor on both the inlet and obturator outlet views, the screw length is measured with a similar-sized K-wire.

If a non-cannulated screw is to be used for fixation, drilling is performed directly from the optimal entry point without a K-wire, and it is confirmed fluoroscopically that the drill remains within the corridor. After drilling, the length of the corridor is measured. In cases where compression is planned during screw insertion, a washer can be used to prevent the screw head from sinking into the cortical bone.

**Fig. 6:**
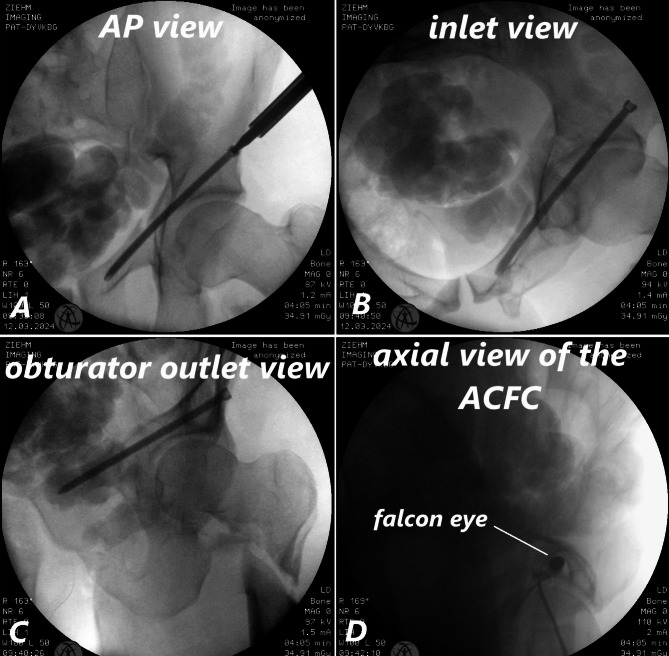
Traditional fluoroscopic views (AP, inlet, obturator outlet) confirming screw placement (**A-C**). Axial ACFC view with the “Falcon Eye” sign verifying the screw position (**D**)

Once the corridor is prepared for the screw, the appropriate screw is inserted, and its position is confirmed using AP, inlet, and obturator outlet views of the pelvis (Fig. 6A–C). If desired, the surgeon can also verify the screw position in the axial view. In this view, we refer to the specific appearance of the screw within the corridor as the ‘Falcon Eye.’ The presence of the ‘Falcon Eye’ in the axial view further confirms the correct placement of the screw within the corridor (Fig. 6D). While the ‘Falcon Sign’ refers to the characteristic anatomical shape observed on axial fluoroscopy before screw placement, the ‘Falcon Eye’ describes the fluoroscopic appearance of the screw correctly positioned within the corridor.

## Discussion

The anterior column fixation corridor (ACFC) of the acetabulum is an osseous pathway extending from the pubic symphysis to the supraacetabular lateral iliac cortex, encompassing the superior pubic ramus and the anterior acetabular wall. The ACFC can be utilized for both antegrade and retrograde percutaneous screw placement [9]. However, due to the complex pelvic anatomy, screw placement in the anterior column presents challenges. Although intraoperative advanced imaging systems and computer-assisted navigation systems increase the accuracy of screw placement, these methods are expensive and not available in every center [10–13]. Therefore, the development of fluoroscopic techniques, which are more affordable and available in nearly every operating room, remains crucial [3, 13].

When placing a screw into the ACFC, the correct selection of the entry point and proper angulation of the screw ensure optimal placement without breaching the corridor [5]. The ideal screw should be centrally positioned in the narrowest part of the corridor and should extend along the entire corridor without exceeding its boundaries [11]. The antegrade screw entry area of the ACFC is wider than the retrograde entry area; while this relatively facilitates screw placement, it can make it more difficult to determine the optimal entry point for fully filling the corridor [14].

In this study, a technique for determining the optimal antegrade entry point of the ACFC and the method for screw placement were described step by step, utilizing only fluoroscopy in the supine position that can be easily applied in every operating room. The detailed methodology of the technique was demonstrated on both a sawbone model and a real acetabular fracture case. Additionally, the feasibility of the technique was investigated using Synapse 3D software, which provides simulations that closely resemble actual fluoroscopic images by converting 3D tomographic images into 2D X-ray images.

When reviewing the literature, we find that very few studies have been conducted focusing on the optimal entry point of the ACFC. Dupuis et al. performed a study using 19 pelvic virtual models, placing a total of 157 screws from different angles. Their aim was to investigate whether a universal entry point could be used for every patient. However, they did not identify a universal optimal entry point and emphasized the importance of using 3D virtual software for preoperative planning to determine the optimal entry point for each patient [5]. Recently, Bai et al. conducted a study where they virtually placed screws into the ACFC one by one, calculating the distances of the antegrade entry points to the roof of the acetabulum, the anterior superior iliac spine, and the greater sciatic notch. They highlighted that there was no universal fixed point that could be used for every patient, and that customizing screw parameters through specific measurements could improve the accuracy of screw placement [15]. The software proposed by the authors provides significant information about personalized screw parameters in preoperative planning; however, it does not help in identifying the optimal entry point for the ACFC in surgical practice [13, 16]. In determining the optimal entry point for the ACFC, pelvic AP view, inlet, and obturator outlet views are generally used. However, identifying the correct entry point may require multiple fluoroscopic images, which can lead to excessive radiation exposure [17]. The axial view of the ACFC and patient-specific optimal antegrade entry point (OAEP) may offer some advantages compared to traditional fluoroscopic methods. Firstly, this technique can easily identify the optimal points for guide pins and screws, potentially shortening the surgical time and reducing the amount of radiation exposure associated with traditional methods for OAEP identification. Additionally, it may reduce the risk of corridor breach caused by eccentric screw placement [9, 13, 18].

A review of the literature reveals that there are few studies investigating screw placement in the ACFC. These studies generally focus on the anthropometric measurements of the ACFC and do not address the fluoroscopic visualization of the OAEP in surgical practice [14, 15, 19–22].

In a study by Zheng et al. [13], retrograde screws were placed in the ACFC on 8 cadavers. They axially visualized the retrograde entry point of the ACFC and placed a total of 16 pins on both the right and left sides.

In their study Feng et al. [14], conducted in 2015 on 5 cadaver hemipelvises, the retrograde entry point of the ACFC was visualized axially, and retrograde screws were placed. However, the fluoroscopy position they described did not seem applicable in normal clinical practice. In their application, the fluoroscopy machine was positioned on the cranial side of the cadaver, which posed a risk of interference with the anesthesia unit when adapted to clinical practice, necessitating further development of the technique. In 2017, the same authors positioned the fluoroscopy machine at the caudal end of the operating table to improve the applicability of their previous technique [23]. This time, the C-arm of the fluoroscopy remained under the patient’s lower extremities. To facilitate the technique’s applicability, they described different positions for both lower extremities. However, this technique raised several questions. The first question was how to position the C-arm under the patient’s legs without compromising sterility. The second question concerned how to practically achieve these fluoroscopic transitions when it was necessary to verify the screw position using traditional fluoroscopic methods. Therefore, although they had demonstrated the fixation corridor axially using healthy human models in fluoroscopy, this technique does not seem very feasible in practice, as the authors have not demonstrated it in a real case.

The axial fluoroscopic imaging technique for the ACFC described in this study is an inexpensive and accessible method that can be applied in any operating room, allowing for the identification of a completely patient-specific OAEP without the need for expensive software and imaging systems. Additionally, it facilitates the identification of the OAEP compared to traditional fluoroscopic techniques and may reduce fluoroscopic radiation exposure. Furthermore, the applicability of the technique in the supine position makes it suitable for most pelvic and acetabular surgeries.

Quercetti et al. defined the axial retrograde optimal screw entry point for screw placement in the ACFC on 10 cadavers. They placed a total of 20 retrograde pins, 10 on each side of the cadavers. They successfully placed 18 pins (%90) without breaching the corridor [24].

In 2020, Vaidya et al. [25] shared the results of 16 cases in which they performed screw placement using the fluoroscopic axial view of the ACFC in the lateral decubitus position. Their aim was to define a new technique to obtain suitable imaging for safely placing the anterior column screw while the patient is in the lateral decubitus position. This study was the only one demonstrating screw placement using the axial fluoroscopic imaging technique of the ACFC in clinical practice. However, their technique was not applicable in most clinical scenarios requiring an anterior approach in the supine position.

To the best of our knowledge, the current study is the first to describe the screw placement technique using axial fluoroscopic imaging of the ACFC in the supine position in clinical practice. Unlike the study by Vaidya et al., this study investigates the technique for axially visualizing the ACFC in the supine position. The applicability of the technique in the supine position makes it particularly feasible in most clinical scenarios that require an anterior approach.

The primary outcomes of this study was the determination of the axial fluoroscopic view of the ACFC and the optimal entry point. In all patients included in the study, regardless of gender, the ACFC and OAEP were shown axially via fluoroscopy. This supports the notion that the ACFC and OAEP can be visualized axially in clinical practice with an appropriate fluoroscopic setup. However, while the fluoroscopic simulations were realistic, it should be noted that the pelvises obtained from healthy individuals may not fully reflect real surgical conditions. Factors such as the patient’s BMI, fracture configuration, and the temporary instruments used for reduction can complicate this visualization.

The secondary outcomes of the study included the investigation of gender-specific anatomical differences and the analysis of measurements taken during screw placement. These analyses included the diameter and length of the ACFC, as well as the necessary Coronal plane inclination (CPI) and sagittal plane inclination (SPI) for fluoroscopic measurements. Measuring the thickness and length of the ACFC provides important information that can assist in selecting an ideal size and thickness for screws that can be placed in the corridor during surgical practice. These data are presented in detail in Table 1. Significant gender differences in ACFC dimensions have been reported in previous studies. Ruzon et al. [20], Bai et al. [15], and Ochs et al. [19] demonstrated that both the ACFC-R and ACFC-L are smaller in females compared to males. Consistent with these findings, our study, based on 500 healthy CT scans, revealed that the ACFC is both narrower and shorter in females than in males. This indicates that, during placement of screws into the ACFC) the risk of screw perforation through the cortical walls may be higher in female patients due to the narrower and shorter corridor. Therefore, more precise preoperative planning may be particularly important in female cases. Taking these anatomical differences into account during surgery—together with careful preoperative assessment—may help minimize the risk of cortical breach.

When examining the studies conducted to reveal the relationship of the ACFC or the screws placed in the anterior column with different planes, Chen et al. [26] described the three-dimensional position of the ACFC axis in relation to the axial, coronal, and sagittal planes using CT scans. Ebraheim et al. [27] utilized the outer table of the iliac wing as a reference for screw trajectory in antegrade screw placement, identifying its inclination relative to the sagittal and coronal planes. However, these anatomical planes are not commonly used in daily clinical practice, making them challenging to visualize [20].

Unlike these studies, our research measured the fluoroscopic inclinations necessary for the axial visualization of the ACFC and OAEP in clinical practice. For this purpose, the angle (CPI) between the ACFC axis and the axial plane was measured in the fluoroscopic pelvis AP view, and the angle (SPI) between the ACFC axis and the frontal plane was measured in the fluoroscopic pelvis lateral view. The measurements obtained by referencing the fluoroscopic planes can provide the surgeon with significant technical information during the fluoroscopy setup in clinical practice.

This study has several limitations. First, the data were obtained from a single institution, which may limit the generalizability of the findings to other ethnic or geographic populations. Second, although the technique was validated in a simulated environment using healthy pelvic models, real surgical conditions may introduce additional challenges. Since the technique relies heavily on the fluoroscopic visualization of anatomical landmarks, factors such as complex, comminuted fracture patterns, soft tissue interference, increased body mass index (BMI) leading to reduced image clarity due to greater soft tissue thickness, and the presence of reduction instruments may hinder the clear identification of these landmarks during surgery, thereby limiting the applicability of the technique. At present, the method appears more feasible for minimally displaced and simple anterior column fractures. Further clinical research is necessary to assess how this fluoroscopic technique performs under real-world surgical conditions and to determine its applicability across different fracture patterns.

## Conclusion

The technique for axial visualization of the anterior column fixation corridor using 3D fluoroscopic simulation demonstrates the potential to accurately identify patient-specific entry points and simulate screw placement within the corridor boundaries. As a simulation-based study, it offers a practical and accessible framework that may support fluoroscopy-assisted anterior column fixation. However, the findings should be interpreted within the context of a virtual environment. These findings lay the groundwork for future studies aimed at translating this simulation-based technique into routine clinical practice and evaluating its potential benefits in terms of surgical accuracy, safety, and efficiency.

## Electronic supplementary material

Below is the link to the electronic supplementary material.

Supplementary Material 1

## Data Availability

No datasets were generated or analysed during the current study.

## Declarations

### Declaration artificial intelligence

The ChatGPT-4 software has been used for the English editing of the article. After using the ChatGPT-4 software, the authors reviewed and edited the content as needed and take full responsibility for the content of the publication.

### Clinical Trials Registration

The study has been registered on ClinicalTrials.gov with the ID number: NCT06634251.
